# Bcl-2 overexpression blocks caspase activation and downstream apoptotic events instigated by photodynamic therapy

**DOI:** 10.1038/sj.bjc.6690017

**Published:** 1999-09-24

**Authors:** D J Granville, H Jiang, M T An, J G Levy, B M McManus, D W C Hunt

**Affiliations:** QLT PhotoTherapeutics, 520 West 6th Avenue, Vancouver, BC V5Z 4H5, Canada; Department of Pathology and Laboratory Medicine, University of British Columbia, 2211 Westbrook Mall, Vancouver, BC V6T 2B5, Canada; Department of Microbiology and Immunology, Faculty of Science, University of British Columbia, 300-6174 University Boulevard, Vancouver, BC V6T 1W5, Canada

**Keywords:** apoptosis, photodynamic therapy, Bcl-2, caspase, resistance, leukaemic cells

## Abstract

Treatment with the photosensitizer benzoporphyrin derivative monoacid ring A (BPD-MA, verteporfin) followed by irradiation with visible light induces apoptosis in human acute myelogenous leukaemia HL-60 cells. Photoactivation of BPD-MA induces procaspase 3 (CPP32/Yama/apopain) and procaspase 6 (Mch2) cleavage into their proteolytically active subunits in these cells. The Bcl-2 proto-oncogene product has been shown to protect cells from a number of proapoptotic stimuli. In the present study, the influence of Bcl-2 overexpression on cellular resistance to photoactivation of BPD-MA was studied. Overexpression of Bcl-2 in HL-60 cells prevented apoptosis-related events including caspase 3 and 6 activation, poly(ADP-ribose) polymerase cleavage and the formation of hypodiploid DNA produced by BPD-MA (0–200 ng ml^−1^) and light. However, Bcl-2 overexpression was less effective at preventing cell death that occurred after photoactivation at high levels (50–100 ng ml^−1^) compared with lower doses (10–25 ng ml^−1^) of BPD-MA. These results indicate that caspase 3 and 6 activation and their regulation by Bcl-2 may play important roles in photodynamic therapy (PDT)-induced cell killing. © 1999 Cancer Research Campaign


*British Journal of Cancer* (1999) **79**(1), 95–100
© 1999 Cancer Research Campaign

### Bcl-2 overexpression blocks caspase activation and downstream apoptotic events instigated by photodynamic therapy

**DJ Granville[1,2], H Jiang[1], MT An[1], JG Levy[1,3], BM McManus[2] and DWC Hunt[1,2]**

[1]QLT PhotoTherapeutics, 520 West 6th Avenue, Vancouver, BC V5Z 4H5, Canada; [2]Department of Pathology and Laboratory Medicine, University of British Columbia, 2211 Westbrook Mall, Vancouver, BC V6T 2B5, Canada; [3]Department of Microbiology and Immunology, Faculty of Science, University of British Columbia, 300–6174 University Boulevard, Vancouver, BC V6T 1W5, Canada

**Summary** Treatment with the photosensitizer benzoporphyrin derivative monoacid ring A (BPD-MA, verteporfin) followed by irradiation with visible light induces apoptosis in human acute myelogenous leukaemia HL-60 cells. Photoactivation of BPD-MA induces procaspase 3 (CPP32/Yama/apopain) and procaspase 6 (Mch2) cleavage into their proteolytically active subunits in these cells. The Bcl-2 proto-oncogene product has been shown to protect cells from a number of proapoptotic stimuli. In the present study, the influence of Bcl-2 overexpression on cellular resistance to photoactivation of BPD-MA was studied. Overexpression of Bcl-2 in HL-60 cells prevented apoptosis-related events including caspase 3 and 6 activation, poly(ADP-ribose) polymerase cleavage and the formation of hypodiploid DNA produced by BPD-MA (0–200 ng ml$^{-1}$) and light. However, Bcl-2 overexpression was less effective at preventing cell death that occurred after photoactivation at high levels (50–100 ng ml$^{-1}$) compared with lower doses (10–25 ng ml$^{-1}$) of BPD-MA. These results indicate that caspase 3 and 6 activation and their regulation by Bcl-2 may play important roles in photodynamic therapy (PDT)-induced cell killing.

**Keywords:** apoptosis; photodynamic therapy; Bcl-2; caspase; resistance; leukaemic cells,

Photodynamic therapy (PDT) is an approved clinical technique for the treatment of malignancies. PDT involves the topical or systemic application of a photosensitizing agent followed by illumination at a specific ‘activating’ wavelength of light (Gomer et al, 1988; Jamieson et al, 1993; Kick et al, 1995). Light activation of photosensitizers is believed to kill cells by catalysing the production of reactive oxygen intermediates (Gomer et al, 1988). Our laboratory is interested in the biological activity of the chlorin-type photosensitizer, benzoporphyrin derivative monoacid ring A (BPD-MA) (Richter et al, 1987). BPD-MA accumulates to higher levels in leukaemic cells compared with normal blood mononuclear cells (Jamieson et al, 1990, 1993). Further, using light-activated BPD-MA, in vitro experiments have demonstrated a selective killing of leukaemic cells over normal haematopoietic progenitor cells (Jamieson et al, 1990; Gluck et al, 1996). Electron microscopy, histological and biochemical studies have shown that PDT with a variety of photosensitizers induces apoptosis in different cell types (Gomer et al, 1988; Zaidi et al, 1993; Tajiri et al, 1996; Granville et al, 1997). Treatment of human promyelocytic leukaemia HL-60 cells with BPD-MA and light rapidly induces DNA fragmentation, caspase activation and apoptotic cell death (Granville et al, 1997, 1998*a*). However, it cannot be ruled out that PDT might also inflict damage to cells which promote passive necrotic cell death (Noodt et al, 1996).

*Received 23 January 1998*
*Revised 1 June 1998*
*Accepted 6 June 1998*

*Correspondence to:* D Granville at QLT PhotoTherapeutics, 520 West 6th Avenue, Vancouver, BC V5Z 4H5, Canada

It is now established that proteolytic cleavage of key cellular substrates is a fundamental biochemical event underlying the apoptotic process (Casciola-Rosen et al, 1996). One of the most intensively studied of these proteases, caspase 3 (previously termed CPP32/Yama/apopain), normally resides in the cytosolic fraction of cells as an inactive precursor and is proteolytically activated in cells undergoing apoptosis (Schlegel et al, 1996). Increasing numbers of caspase 3 substrates have been identified and include poly(ADP-ribose) polymerase (PARP) (Nicholson et al, 1995), sterol regulatory element binding proteins (Wang et al, 1996), the U1-associated 70-kDa protein (Casciola-Rosen et al, 1996), DNA-dependent protein kinase (Casciola-Rosen et al, 1996) and DNA fragmentation factor (DFF) (Liu et al, 1997). Recent studies have demonstrated that caspase 3 activation and DNA fragmentation are directly linked through the caspase-3-mediated cleavage of DNA fragmentation factor (DFF) (also known as inhibitor of caspase-activated deoxyribonuclease, ICAD), a cytosolic factor which binds to and inhibits the activity of an endonuclease (caspase-activated nuclease, CPAN or caspase-activated deoxyribonuclease, CAD) directly responsible for DNA fragmentation during apoptosis (Liu et al, 1997; Enari et al, 1998; Halenbeck et al, 1998, Sakahira et al, 1998). This endonuclease, CAD/CPAN, is only activated during apoptosis and is, therefore, believed to be responsible for DNA fragmentation (Halenbeck et al, 1998; Sakahira et al, 1998). We have shown that caspase-3-dependent cleavage of DFF occurs in PDT-treated HL-60 (Granville et al, 1998*b*) and HeLa cells (Carthy et al, 1998).

Caspase 6 (Mch2) is also mobilized in response to certain apoptotic stimuli (Fernandes-Alnemri et al, 1995; Orth et al, 1996; Srinivasula et al, 1996). Caspase 6 is believed to be responsible for nuclear lamin cleavage which may contribute to the nuclear degradation observed during apoptosis (Orth et al, 1996; Srinivasula et al, 1996; Takahashi et al, 1996).

One of the most studied cell survival genes is Bcl-2 (Gajewski and Thompson, 1996; Reed, 1997). Bcl-2 was discovered as an overexpressed protein in human B-cell lymphomas arising as a result of a t(14;18) chromosomal translocation (Pegoraro et al, 1984; Tsujimoto et al, 1985). The overexpression of Bcl-2 has been shown to protect different cell types against apoptosis induced by such diverse stimuli as viral infection, hypoxia, ionizing radiation or chemotherapeutic agents (Shimizu et al, 1995; Gajewski and Thompson, 1996; Ibrado et al, 1996, 1997; Reed, 1996). Bcl-2 overexpression in Chinese hamster ovary cells provided partial protection against the loss of clonogenicity produced by PDT using phthalocyanine Pc4 (He et al, 1996), supporting a role for Bcl-2 in the regulation of PDT-induced apoptosis. Several members of the Bcl-2 family have been identified in mammals: Bcl-2, Bcl-$X_L$, A1/Bfl-1, Bcl-w, Nr13 and Mcl-1 serve to inhibit apoptosis, whereas Bax, Bik, Bak, Bad and Bcl-$X_S$ promote apoptosis (Reed, 1996; Nagata, 1997). Members of the Bcl-2 family have been shown to homo- or heterodimerize with one another to either antagonize or enhance the function of the other (Yang et al, 1995; Reed, 1996). In vivo and in vitro studies showed that Bcl-2 provides protection against apoptosis in the absence of protein translation, suggesting that it does not exert its protective effect through gene regulation (Rowan and Fisher, 1997). Bcl-2 is an integral membrane protein that is situated primarily on the outer membranes of mitochondria, endoplasmic reticulum and nuclei (Nagata, 1997; Yang et al, 1997). In some systems, Bcl-2 regulates intracellular $Ca^{2+}$ levels and prevents the loss of mitochondrial membrane potential produced by proapoptotic stimuli (Rowan and Fisher, 1997). Recent evidence suggests that Bcl-2 may act as an ion channel and regulate the release of cytochrome *c* from the mitochondria, an event that may be necessary for caspase 3 activation (Kluck et al, 1997; Yang et al, 1997).

We have previously demonstrated that caspase 3, but not caspase 1, is activated leading to PARP and DNA-PK cleavage in HL-60 cells treated with cytotoxic levels of BPD-MA and light (Granville et al, 1997). The present report shows that although Bcl-2 overexpression effectively blocked caspase 3 and caspase 6 activation as well as hypodiploid DNA formation produced by PDT, Bcl-2 had little or no effect on cell death induced by the photoactivation of BPD-MA at high drug doses but did offer a degree of protection against killing at lower levels of the photosensitizer as determined by 3-day culture studies.

### MATERIALS AND METHODS

### Reagents and cell culture

Liposomally formulated BPD-MA was from QLT PhotoTherapeutics (Vancouver, BC, Canada). All antibodies were purchased from Santa Cruz Biotechnology (Santa Cruz, CA, USA). All other drugs and chemicals, unless specified, were obtained from Sigma Chemical (St. Louis, MO, USA). The transfected human promyelocytic leukaemia HL-60/neo and HL-60/Bcl-2 cell lines were generously provided by Dr Kapil Bhalla (Emory University School of Medicine, Atlanta, GA, USA). Detailed methods used to generate these clones have been previously described (Ibrado et al, 1996). The HL-60/Bcl-2 cells used in this study contain a fivefold higher level of Bcl-2 than the HL-60/neo control cells, whereas Bcl-$X_L$ and Bax levels are comparable for both transfectants (Ibrado et al, 1996). Cells were maintained in RPMI-1640 medium supplemented with 10% heat-inactivated fetal bovine serum (FBS), 4 mM L-glutamine, 1 mM sodium pyruvate, 1 mM Hepes, penicillin (100 U ml$^{-1}$) and streptomycin (100 μg ml$^{-1}$) (Gibco, Burlington, Ontario, Canada) and G418 (1 mg ml$^{-1}$) (Geneticin; Life Technologies, Grand Island, NY, USA).

### PDT and inhibitor treatment of cells

For photodynamic studies, cells were incubated for a total of 60 min at 37°C with BPD-MA (0–200 ng ml$^{-1}$) in RPMI containing 10% FBS. After incubation with BPD-MA, cells were exposed to fluorescent red light (620–700 nm) delivered at 5.6 mW cm$^{-2}$ to give a total dose of 2 J cm$^{-2}$. For caspase 3 inhibition studies, Z-DEVD-fmk (Enzyme Systems Products, Dublin, CA, USA) was added to the cells to give final concentrations of 10, 20 or 25 μM for the final 30 min before photoactivation.

### Analysis of DNA status

The propidium iodide (PI) fluorescence analysis procedure was used to detect changes in the status of cellular DNA (Darzynkieicz et al, 1992; Telford et al, 1994). At 3 h, after PDT, $1 \times 10^6$ cells were washed twice with ice-cold PBS then permeabilized and fixed in 80% ethanol at 4°C for 1 h. Cells were washed twice in ice-cold PBS and treated with RNAase (5 U ml$^{-1}$, DNAase-free) and stained with PI (50 μg ml$^{-1}$) in PBS. Samples were analysed by flow cytometry. The percentage of cells containing hypodiploid levels of DNA was calculated from single parameter flow cytometry for PI fluorescence (Telford et al, 1994) using an Epics XL flow cytometer (Coulter Electronics, Hialeah, FA, USA).

### Preparation of cellular protein extracts

To prepare cell lysates, cells were initially washed twice with ice-cold PBS. Cell pellets were treated with 1 ml of lysis buffer [1% Nonidet P-40 detergent (NP-40), 20 mM Tris, pH 8, 137 mM sodium chloride, 10% glycerol] supplemented with 1 mM phenylmethylsulphonyl fluoride (PMSF), aprotinin (0.15 U ml$^{-1}$) and 1 mM sodium orthovanadate for 20 min on ice. Lysates were centrifuged for 10 min at 15 800 ***g*** at 4°C. Protein concentrations of cell extracts were determined with the Pierce BCA protein assay (Pierce, Rockford, IL, USA).

### Protease assay

To evaluate caspase 3 activity, cell lysates were prepared 1 h after their respective treatment. Assays were performed in 96-well microtitre plates by incubating 25 μl (10 μg protein) of cell lysate in 125 μl of reaction buffer (1% NP-40, 20 mM tris-HCl, pH 7.5, 137 mM sodium chloride, 10% glycerol) containing the caspase-3 substrate [Acetyl-Asp-Glu-Val-Asp-amino-4-methyl-coumarin (Ac-DEVD-AMC)] (Calbiochem, Cambridge, MA, USA) at 100 μM. Lysates were incubated at 37°C for 16 h and fluorescence levels were determined using a CytoFluor 2350 (PerSeptive Biosystems, Burlington, ON, USA) set at excitation and emission wavelengths of 380 nm and 460 nm respectively.

© Cancer Research Campaign 1999

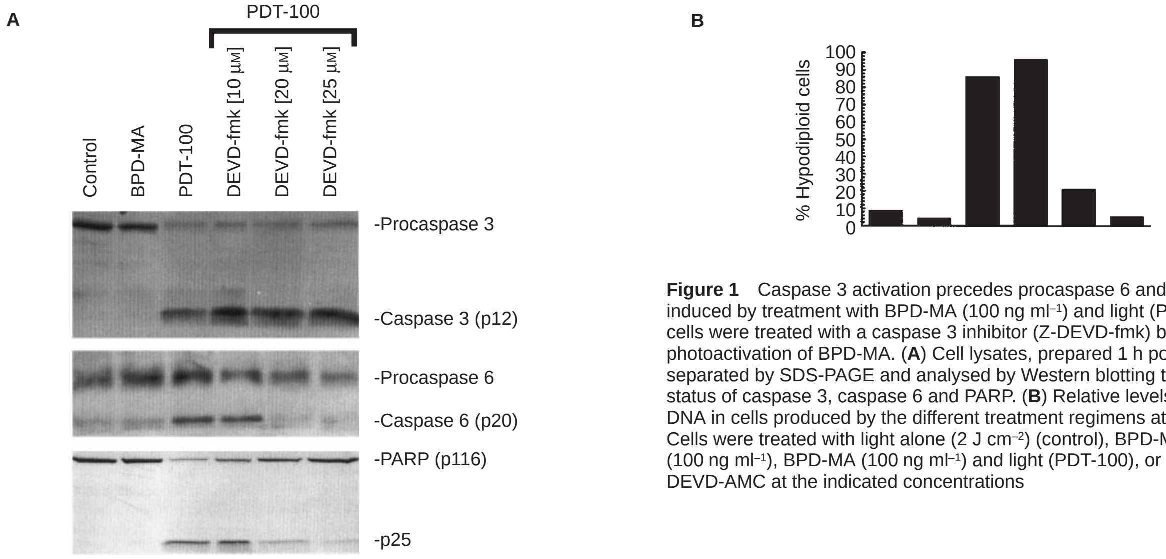

**Figure 1** Caspase 3 activation precedes procaspase 6 and PARP cleavage induced by treatment with BPD-MA (100 ng $ml^{-1}$) and light (PDT-100). HL-60 cells were treated with a caspase 3 inhibitor (Z-DEVD-fmk) before photoactivation of BPD-MA. (**A**) Cell lysates, prepared 1 h post-PDT, were separated by SDS-PAGE and analysed by Western blotting to determine the status of caspase 3, caspase 6 and PARP. (**B**) Relative levels of hypodiploid DNA in cells produced by the different treatment regimens at 3 h post-PDT. Cells were treated with light alone (2 J $cm^{-2}$) (control), BPD-MA alone (100 ng $ml^{-1}$), BPD-MA (100 ng $ml^{-1}$) and light (PDT-100), or PDT-100 and Z-DEVD-AMC at the indicated concentrations

### Immunoblot analysis

Detergent-soluble proteins (30 μg) were separated by sodium dodecyl sulphate polyacrylamide gel electrophoresis (SDS-PAGE) in 12% gels, under reducing conditions (Laemmli, 1970). Proteins were transferred to nitrocellulose membranes at 100 V for 1 h. Membranes were blocked for 30 min at room temperature with 5% skimmed milk powder in PBS-0.05% Tween 20 (PBS-T). Membranes were incubated for 45 min using the following polyclonal antibodies: goat-anti-PARP, goat-anti-CPP32, goat-anti-Mch2 or rabbit-anti-Bcl-2 at 1 μg $ml^{-1}$. Membranes were then probed with anti-goat IgG-HRP or anti-rabbit IgG-HRP (1 : 5000) in PBS-T with 1% skimmed milk powder for 30 min at room temperature. Membranes were rinsed twice in PBS-T, followed by three 15-min washes with PBS-T. Proteins were detected using the enhanced chemiluminescence detection system (Amersham, Arlington Heights, IL, USA) and bands visualized by autoradiography.

### Assessment of cell viability using MTT

To assess cell viability, $5 \times 10^4$ cells were loaded into eight replicate wells (0.2 ml per well) of 96-well microtitre plates. After 3, 24, 48 or 72 h, 10 μl of a MTT solution (5 mg $ml^{-1}$) was added to each well (Mosmann, 1983). Cells were incubated for a further 1 h at 37°C. The reaction was stopped by the addition of 150 ml of acidified isopropanol. The degree of colour development was analysed with an automated densitometer microtitre plate reader (Dynatech, Hamilton, VA, USA) using a 590-nm filter.

### RESULTS

### Caspase 6 activation occurs downstream of caspase 3

To assess the involvement of caspases in PDT-induced apoptosis, wild-type HL-60 cells were treated with different amounts of the caspase 3 inhibitor Z-DEVD-fmk before BPD-MA photoactivation. The presence of Z-DEVD-fmk did not affect PDT-induced caspase-3 cleavage and the appearance of the 12-kDa cleavage product at the concentrations tested (Figure 1). However, when the membrane was probed with either anti-caspase 6 or anti-PARP antibodies, it was evident that PDT-induced cleavage of these proteins was greatly reduced at a 25 μM concentration of Z-DEVD-fmk. The PDT-induced increase in hypodiploid DNA was also blocked at this concentration of the tetrapeptide inhibitor. A lower concentration of Z-DEVD-fmk (10 μM) did not block the cleavage of procaspase 6 or PARP, nor affect the appearance of hypodiploid levels of DNA (Figure 1). These results demonstrate that caspase 6 is activated by PDT and may occur downstream of caspase-3 processing.

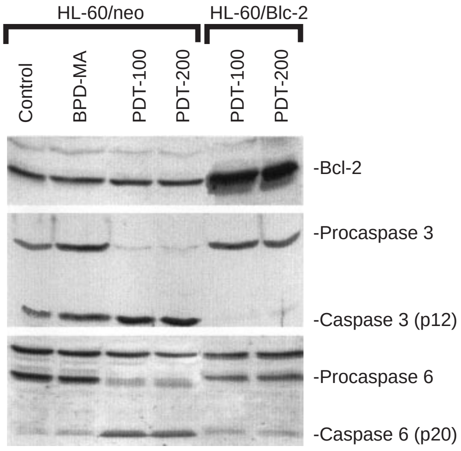

**Figure 2** Overexpression of Bcl-2 in HL-60 cells blocks the processing of procaspase 3 and procaspase 6 induced by treatment with BPD-MA and light. Cells were lysed 1 h after photosensitization. Cell lysates were separated by SDS-PAGE followed by Western immunoblotting. Cells were treated with light alone (2 J $cm^{-2}$) (control), BPD-MA alone (100 ng $ml^{-1}$) and light (PDT-100), or BPD-MA (200 ng $ml^{-1}$ and light (PDT-200)

### Overexpression of Bcl-2 blocks caspase 3 and caspase 6 cleavage

To assess the influence of Bcl-2 overexpression and susceptibility to photodynamic killing, HL-60/neo or HL-60/Bcl-2 cells were incubated with or without BPD-MA and then exposed to light. Cells were lysed 1 h after light activation of BPD-MA. The status of Bcl-2, caspase 3 and caspase 6 in cytosolic extracts was

*© Cancer Research Campaign 1999*

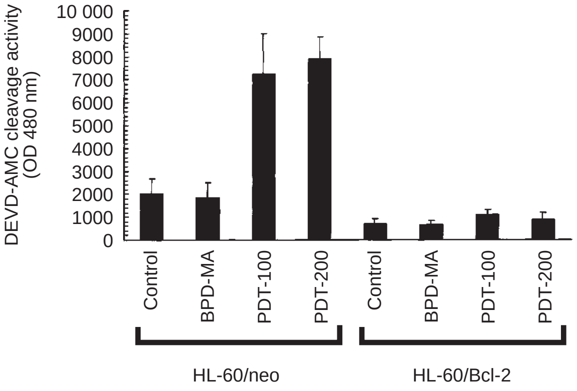

**Figure 3** Overexpression of Bcl-2 blocks the induction of DEVD-ase activity associated with PDT. AT 1 h after treatment, HL-60/neo (■) or HL-60/Bcl-2 (□) cell lysates were prepared and assayed for their capacity to cleave a fluorescently labelled caspase 3 substrate (Ac-DEVD-AMC). Cytosolic extracts were taken from untreated cells (control) or cells treated with BPD-MA (100 ng ml$^{-1}$) alone, BPD-MA (100 ng ml$^{-1}$) and light (2 J cm$^{-2}$) (PDT-100) or BPD-MA (200 ng ml$^{-1}$) and light (2 J cm$^{-2}$) (PDT-200). Error bars represent standard deviations (s.d.) of the mean values obtained in three experiments using replicates of five for each treatment sample

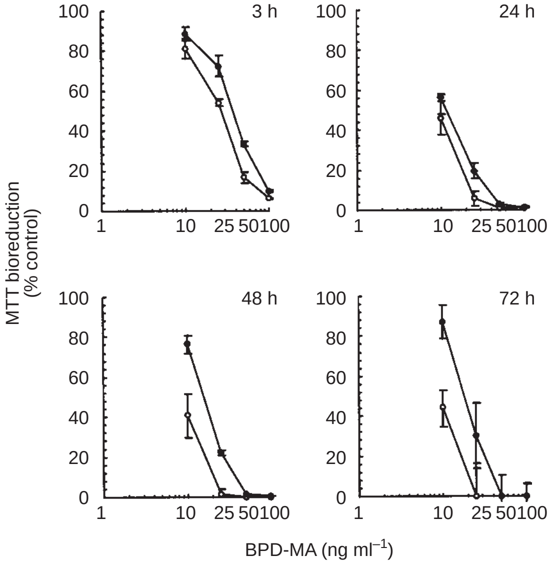

**Figure 5** Overexpression of Bcl-2 provides protection against PDT-induced cell death only for cells treated with concentrations of BPD-MA of 25 ng ml$^{-1}$ or less. HL-60/neo (○) and HL-60/Bcl-2 (●) cells were incubated with BPD-MA (0–100 ng ml$^{-1}$) and exposed to light 1 h later. Cell viability was assayed 3, 24, 48 or 72 h later by measuring the bioreduction of MTT. Data are expressed as a percentage of the result obtained with untreated control cells. Error bars represent the s.d. of mean values from two experiments using eight replicates for each treatment

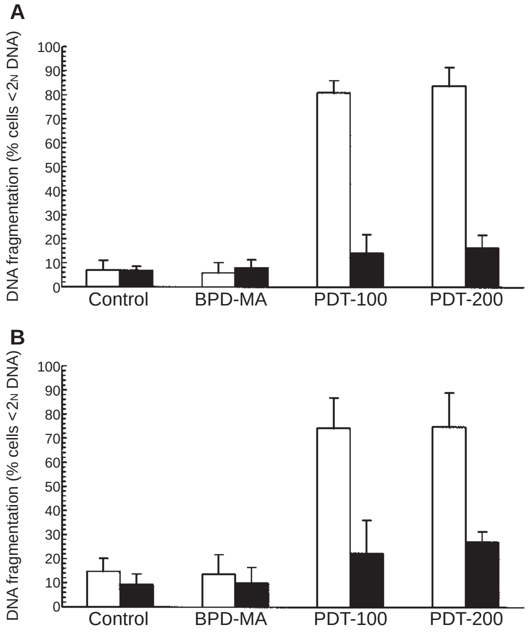

**Figure 4** Overexpression of Bcl-2 in HL-60 cells blocks PDT-induced hypodiploid DNA formation. HL-60/neo (□) and HL-60/Bcl-2 (■) cells were assessed for the presence of hypodiploid DNA by PI staining, (**A**) 3 h or (**B**) 24 h after the indicated treatments. Cells were treated with medium (control), BPD-MA (100 ng ml$^{-1}$), BPD-MA (100 ng ml$^{-1}$) and light (2 J cm$^{-2}$) (PDT-100), or BPD-MA (200 ng ml$^{-1}$) and light (2 J cm$^{-2}$) (PDT-200). Mean values with s.d. for three independent experiments are shown determined by Western immunoblotting. The control Bcl-2-transfected cells exhibited the expected greater band density of Bcl-2 (Figure 2). The electrophoretic status of Bcl-2 was unaffected by PDT treatment of either cell type (HL-60/neo or HL-60/Bcl-2). For cells treated with BPD-MA and light, caspase 3 and caspase 6 cleavage was evident for HL-60/neo cell lysates, but not in the HL-60/Bcl-2 cell extracts (Figure 2). These results suggest that Bcl-2 may indirectly or directly influence caspase 3 and caspase 6 activation produced by PDT.

### Overexpression of Bcl-2 blocks caspase 3-like protease activity

Cell lysates were assayed for their capacity to cleave a fluorescently labelled caspase 3 substrate (Ac-DEVD-AMC) (Figure 3). Lysates prepared from HL-60/neo cells treated with BPD-MA and light exhibited high Ac-DEVD-AMC cleavage activity, whereas lysates from HL-60/Bcl-2 cells treated with the same levels of BPD-MA and light exhibited baseline levels of substrate cleavage. These results further support the observation that overexpression of Bcl-2 blocks activation of caspase 3 instigated by BPD-MA photoactivation.

### Overexpression of Bcl-2 blocks PDT-induced increases in hypodiploid DNA

HL-60/neo and HL-60/Bcl-2 cells were analysed for their DNA status 3 or 24 h after BPD-MA photoactivation (Figure 4). HL-60/neo cells treated with BPD-MA and light exhibited high levels of hypodiploid DNA, suggestive of DNA fragmentation, compared with untreated cells and cells treated with light or BPD-MA alone. However, there was no evidence of PDT-induced DNA fragmentation (cells < 2*n* DNA) for HL-60/Bcl-2 cells treated with BPD-MA and light at 3 h after photoactivation and only a slight increase in hypodiploid DNA by 24 h. These results indicate that overexpression of Bcl-2 in HL-60 cells prevents the appearance of hypodiploid DNA induced by BPD-MA and light.

### Overexpression of Bcl-2 provides partial protection against cell death at low levels of PDT

Our final step was to examine the effect of Bcl-2 overexpression on the viability of cells treated with BPD-MA and light. HL-60/neo and HL-60/Bcl-2 cells were incubated with titrated amounts of BPD-MA and exposed to light 1 h later. Bioreduction of MTT was used to compare the viability of each cell type at 3, 24, 48 and 72 h

 *© Cancer Research Campaign 1999*

after PDT. Overexpression of Bcl-2 provided no observable protection at concentrations of BPD-MA at 50 or 100 ng $ml^{-1}$, as measured by MTT bioreduction over a 72-h period (Figure 5). However, at lower concentrations of BPD-MA (10 or 25 ng $ml^{-1}$), Bcl-2 overexpression did provide a degree of protection against photodynamic inactivation of a small proportion of cells which exhibited recovery over the 72-h test period.

### DISCUSSION

Overexpression of Bcl-2 has been shown to protect many different cancer cell lines from apoptosis induced by a wide variety of chemotherapeutic agents including dexamethasone, etoposide, methotrexate, cisplatin, cyclophosphamide, vincristine and 1-beta-D-arabinofuranosylcytosine (Ara-C) (reviewed in Reed, 1996). It has been observed that Bcl-2 overexpression blocks caspase 3 activation and subsequent PARP cleavage in these systems (Ibrado et al, 1996; Estoppey et al, 1997; Perry et al, 1997). In this study, we evaluated caspase activity, DNA fragmentation (as evidenced by an increase in hypodiploid DNA) and the viability of HL-60/neo or HL-60/Bcl-2 cells to determine whether treatment with cytotoxic levels of BPD-MA and light was regulated in a similar fashion in the setting of Bcl-2 overexpression.

Overexpression of Bcl-2 prevented procaspase 3 and PARP cleavage produced by PDT. Such findings correspond to previous results obtained with these cells in which Ara-C was used as the apoptotic stimulus (Ibrado et al, 1996). Procaspase 6 was also not processed in HL-60/Bcl-2 cells, indicating that Bcl-2 also acts upstream of caspase 6 activation in cells treated with BPD-MA and light. It has been demonstrated that caspase 6 may be responsible for degradation of nuclear lamins during apoptosis (Lazebnik et al, 1995; Neamati et al, 1995; Takahashi et al, 1996). Lamin cleavage may be critical for the degradation of the nuclear envelope during apoptotic cell death (Lazebnik et al, 1995). Studies by others have shown that caspase 3 can directly cleave procaspase 6 (Srinivasula et al, 1996), suggesting that procaspase 6 cleavage does not occur in PDT-treated HL-60/Bcl-2 cells because of the absence of caspase 3 activity. Conversely, a recent report by Thornberry et al (1997) suggests that the optimal amino acid substrate sequence for caspase 6 resembles the activation site in effector caspase proenzymes such as caspase 3, suggesting that caspase 6 may act upstream of caspase 3 (Thornberry et al, 1997). To determine whether caspase 6 activation occurred downstream of caspase 3, wild-type HL-60 cells were incubated with a caspase 3 inhibitor, Z-DEVD-fmk, before photoactivation of BPD-MA. The caspase 3 inhibitor did not block the appearance of the 12-kDa subunit of caspase 3, as produced by PDT, but did prevent the cleavage of procaspase 6 and PARP indicating that procaspase 6 cleavage is probably downstream of caspase 3 activation.

Cytosolic extracts prepared from HL-60/Bcl-2 cells exhibited minimal cleavage of the Ac-DEVD-AMC substrate in contrast to the HL-60/neo cells treated with the same amounts of BPD-MA and light. Interestingly, untreated HL-60/Bcl-2 cells, or those exposed to the photosensitizer in the absence of light, had a lower constitutive capacity to cleave the Ac-DEVD-AMC substrate than the HL-60/neo cells. This observation could be attributable to the presence of a minor proportion of apoptotic cells within the HL-60/neo cell population exhibiting caspase activity. Background levels of protease activity would be less for HL-60/Bcl-2 cells because overexpression of Bcl-2 inhibits caspase 3 activation (Ibrado et al, 1996).

Several studies have now demonstrated that cytochrome *c* may be necessary for the activation of caspase 3 and that Bcl-2 blocks the release of cytochrome *c* from mitochondria (Kluck et al, 1997; Yang et al, 1997). Furthermore, it has recently been demonstrated that cytochrome *c* is involved in the activation of caspase 9, which is believed to then process caspase 3 (Li et al, 1997). Whether this scenario transpires in PDT-treated cells will be examined in future studies.

For HL-60/Bcl-2 cells treated with BPD-MA and light, cell survival was not detected at photosensitizer concentrations of 50 ng $ml^{-1}$ or greater at 72 h post PDT, suggesting that PDT may circumvent the inhibitory function of Bcl-2. The photodynamic treatment did not override the inhibitory effects of Bcl-2 with respect to caspase 3 and caspase 6 activation because procaspase 3 and 6 cleavage or DNA fragmentation did not occur in the HL-60/Bcl-2 cells that were treated with BPD-MA (100 ng $ml^{-1}$) and light. This would suggest that a parallel biochemical pathway unrelated to caspases may be triggered in cells treated with higher levels of BPD-MA and light. At BPD-MA concentrations of less than 50 ng $ml^{-1}$, a modest protective effect of Bcl-2 overexpression against PDT-mediated cell death was observed in line with observations for other chemotherapeutic treatments (Reed et al, 1996). Although it has been well established by electron microscopy, histological and biochemical analysis that PDT induces apoptosis, we cannot rule out the possibility that PDT inflicts damage to other cell sites promoting passive necrotic cell death at higher drug concentrations (Gomer et al, 1988; Zaidi et al, 1993; Tajiri et al, 1996; Granville et al, 1997). Further understanding towards the mechanisms involved in PDT-induced cell death and how this treatment may be able to overcome the regulatory control of Bcl-2 should provide valuable insights towards the improvement of current cancer treatments.

### ABBREVIATIONS

Ac-DEVD-AMC, acetyl-Asp-Glu-Val-Asp-amino-4-methylcoumarin; BPD-MA, benzoporphyrin monoacid ring A, verteporfin, ICE, interleukin 1 converting enzyme; MTT, 3-(4,4-dimethylthiazol-2-yl)-2,5-diphenyl tetrazolium bromide; PARP, poly(ADP-ribose) polymerase; PDT, photodynamic therapy; PI, propidium iodide; Z-DEVD-fmk, Z-Asp-Glu-Val-Asp-fluoromethylketone.

### ACKNOWLEDGEMENTS

We are grateful to Dr Kapil Bhalla from Emory University School of Medicine (Atlanta, GA, USA) for providing the HL-60/neo and HL-60/Bcl-2 cells. We thank Chris Carthy (University of British Columbia, Canada) for critical review of the manuscript and Brent Rupnow (Stanford University, USA) for providing us with technical advice and critical review of the manuscript.

#### REFERENCES

Carthy CM, Granville DJ, Watson KA, Anderson DR, Wilson JE, Yang DC, Hunt DWC and McManus BM (1998) Caspase activation and specific cleavage of substrates after coxsackievirus B3-induced cytopathic effect in HeLa cells. *J Virol* **72**: 7669–7675

Casciola-Rosen L, Nicholson DW, Chong T, Rowan K, Thornberry NA, Miller D and Rosen A (1996) Apopain/CPP32 cleaves proteins that are essential for cellular repair: a fundamental principle of apoptotic death. *J Exp Med* **183**: 1957–1964

Darzynkieicz Z, Bruno S, Del Bino G, Gorczyca W, Hotz M, Lassota P and Traganos F (1992) Features of apoptotic cells measured by flow cytometry. *Cytometry* **13**: 795–808

© Cancer Research Campaign 1999 

Enari M, Sakahira H, Yokoyama H, Okawa K, Iwamatsu A and Nagata S (1998) A caspase-activated DNase that degrades DNA during apoptosis, and its inhibitor ICAD. *Nature* **391**: 43–50

Estoppey S, Rodriguez I, Sadoul R and Martinou JC (1997) Bcl-2 prevents activation of CPP32 cysteine protease and cleavage of poly(ADP-ribose) polymerase and U1-70 kD proteins in staurosporine-mediated apoptosis. *Cell Death Differ* **4**: 34–38

Fernandes-Alnemri T, Litwack G and Alnemri ES (1995) Mch2, a new member of the apoptotic Ced-3/ICE cysteine protease gene family. *Cancer Res* **55:** 2737–2742

Gajewski TF and Thompson CB (1996) Apoptosis meets signal transduction: elimination of a BAD influence. *Cell* **87**: 589–593

Gluck S, Chadderton A and Ho A (1996) The selective uptake of benzoporphyrin derivative mono-acid ring A results in differential cell kill of multiple myeloma cells in vitro. *Photochem Photobiol* **63**: 846–853

Gomer CJ, Ferrario A, Hayashi N, Rucker N, Szirth B and Murphree A (1988) Molecular, cellular, and tissue responses following photodynamic therapy. *Laser Surg Med* **8**: 450–463

Granville DJ, Levy JG and Hunt DW (1997) Photodynamic therapy induces caspase-3 activation in HL-60 cells. *Cell Death Differ* **4**: 623–629

Granville DJ, Levy JG and Hunt DW (1998*a*) Photodynamic treatment with benzoporphyrin derivative monoacid ring A produces protein tyrosine phosphorylation events and DNA fragmentation in murine P815 cells. *Photochem Photobiol* **67**: 358–362

Granville DJ, Jiang H, An MT, Levy JG, McManus BM and Hunt DW (1998*b*) Overexpression of Bcl-X(L) prevents caspase-3-mediated activation of DNA fragmentation factor (DFF) produced by treatment with the photochemotherapeutic agent BPD-MA. *FEBS Lett* **422**: 151–154

Halenbeck R, MacDonald H, Roulston A, Chen TT, Conroy L and Williams LT (1998) CPAN, a human nuclease regulated by the caspase-sensitive inhibitor DFF45. *Curr Biol* **8**: 537–540

He J, Agarwal ML, Larkin HE, Friedman LR, Xue LY and Oleinick NL (1996) The induction of partial resistance to photodynamic therapy by the protooncogene BCL-2. *Photochem Photobiol* **64**: 845–852

Ibrado AM, Huang Y, Fang G, Liu L and Bhalla K (1996) Overexpression of Bcl-2 or Bcl-$X_L$ inhibits Ara-C-induced CPP32/Yama protease activity and apoptosis of human acute myelogenous leukemia HL-60 cells. *Cancer Res* **56**: 4743–4748

Ibrado AM, Liu L and Bhalla K (1997) Bcl-$X_L$ overexpression inhibits progression of molecular events leading to paclitaxel-induced apoptosis of human acute myeloid leukemia HL-60 cells. *Cancer Res* **57**: 1109–1115

Jamieson CHM, McDonald WN and Levy JG (1990) Preferential uptake of benzoporphyrin derivative by leukemic versus normal cells. *Leukemia Res* **14**: 209–219

Jamieson C, Richter A and Levy J (1993) Efficacy of benzoporphyrin derivative, a photosensitizer, in selective destruction of leukemia cells using a murine tumor model. *Exp Hematol* **21**: 629–634

Kick G, Messer G, Plewig G, Kind P and Goetz AE (1995) Strong and prolonged induction of c-*jun* and c-*fos* proto-oncogenes by photodynamic therapy. *Br J Cancer* **74**: 30–36

Kluck RM, Bossy-Wetzel E, Green DR and Newmeyer DD (1997) The release of cytochrome c from mitochondria: a primary site for Bcl-2 regulation of apoptosis. *Science* **275**: 1132–1136

Laemmli UK (1970) Cleavage of structural proteins during the assembly of the head of bacteriophage T4. *Nature* **227**: 680–685

Lazebnik YA, Takahashi A, Moir RD, Goldman RD, Poirier GG, Kaufmann SH and Earnshaw WC (1995) Studies of the lamin proteinase reveal multiple parallel biochemical pathways during apoptotic execution. *Proc Natl Acad Sci USA* **92**: 9042–9046

Li P, Nijhawan D, Budihardjo I, Srinivasula SM, Ahmad M, Alnemri ES and Wang X (1997) Cytochrome c and dATP-dependent formation of Apaf-1/caspase-9 complex initiates an apoptotic protease cascade. *Cell* **91**: 479–489

Liu X, Zou H, Slaughter C and Wang X (1997) DFF, a heterodimeric protein that functions downstream of caspase-3 to trigger DNA fragmentation during apoptosis. *Cell* **89**: 175–184

Mosmann T (1983) Rapid colorimetric assay for cellular growth and survival: application to proliferation and cytotoxicity assays. *J Immunol Methods* **57**: 55–60

Nagata S (1997) Apoptosis by death factor. *Cell* **88**: 355–365

Neamati N, Fernandez A, Wright S, Kiefer J and McConkey DJ (1995) Degradation of Lamin B1 precedes oligonucleosomal DNA fragmentation in apoptotic thymocytes and isolated thymocyte nuclei. *J Immunol* **154**: 3788–3795

Nicholson WD, Ali A, Thornberry NA, Vaillancourt JP, Ding CK, Gallant M, Gareau Y, Griffin PR, Labelle M and Lazebnik YA (1995) Identification and inhibition of the ICE/CED-3 protease necessary for mammalian apoptosis. *Nature* **376**: 37–43

Noodt BB, Berg K, Stokke T, Peng Q and Nesland JM (1996) Apoptosis and necrosis induced with light and 5-aminolaevulinic acid-derived protoporphyrin IX. *Br J Cancer* **74**: 22–29

Orth K, Chinnaiyan AM, Garg M, Froelich CJ and Dixit VM (1996) The CED-3/ICE-like protease Mch2 is activated during apoptosis and cleaves the death substrate lamin A. *J Biol Chem* **271**: 16443–16446

Pegoraro L, Palumbo A, Erikson J, Falda M, Giovanazzo B, Emanuel BS, Rovero G, Nowell PC and Croce CM (1984) A 14;18 and a 8;14 chromosome translocation in a cell line derived from an acute B-cell leukemia. *Proc Natl Acad Sci USA* **81**: 7166–7170

Perry DK, Smyth MJ, Wang HG, Reed JC, Poirier GG, Obeld LM and Hannun YA (1997) Bcl-2 acts upstream of the PARP protease and prevents its activation. *Cell Death Differ* **4**: 29–33

Reed JC (1996) Mechanisms of Bcl-2 family protein function and dysfunction in health and disease. *Behring Inst Mitt* **97**: 72–100

Reed JC (1997) Double identity for proteins of the Bcl-2 family. *Nature* **387**: 773–776

Reed JC, Miyashita T, Takayama S, Wang HG, Sato T, Krajewski S, Aime-Sempe C, Bodrug S, Kitada S and Hanada M (1996) BCL-2 family proteins: regulators of cell death involved in the pathogenesis of cancer and resistance to therapy. *J Cell Biochem* **60**: 23–32

Richter AM, Kelly B, Chow J, Liu DJ, Towers GH, Dolphin D and Levy JG (1987) Preliminary studies on a more effective phototoxic agent than hematoporphyrin. *J Natl Cancer Inst* **79**: 1327–1332

Rowan S and Fisher DE (1997) Mechanisms of apoptotic cell death. *Leukemia* **11**: 457–465

Sakahira H, Enari M and Nagata S (1998) Cleavage of CAD inhibitor in CAD activation and DNA degradation during apoptosis. *Nature* **391**: 96–99

Schlegel J, Peters I, Orrenius S, Miller DK, Thornberry NA, Yamin T and Nicholson DW (1996) CPP32/Apopain is a key interleukin 1b converting enzyme-like protease involved in Fas-mediated apoptosis. *J Biol Chem* **271**: 1841–1844

Shimizu S, Eguchi Y, Kosaka H, Kamuke W, Matsuda H and Tsujimoto Y (1995) Prevention of hypoxia-induced cell death by Bcl-2 and Bcl-$X_L$. *Nature* **374**: 811–813

Srinivasula SM, Fernandes-Alnemri T, Zangrilli J, Robertson N, Armstrong RC, Wang L, Trapani JA, Tomaselli KJ, Litwack G and Alnemri ES (1996) The ced-3/interleukin 1beta converting enzyme-like homolog mch6 and the lamin-cleaving enzyme mch2alpha are substrates for the apoptotic mediator CPP32. *J Biol Chem* **271**: 27099–27106

Tajiri H, Shinomiya N, Hayakawa A, Matsumoto Y and Yoshida S (1996) Photodynamic therapy-induced rapid cell death by apoptosis in human pancreatic carcinoma transplanted into nude mice. *J Clin Biochem Nutr* **21**: 29–37

Takahashi A, Alnemri ES, Lazebnik YA, Fernandes-Alnemri T, Litwack G, Moir RD, Goldman RD, Poirier GG, Kaufmann SH and Earnshaw WC (1996) Cleavage of lamin A by Mch2a but not CPP32: multiple interleukin 1b-converting enzyme-related proteases with distinct substrate recognition properties are active in apoptosis. *Proc Natl Acad Sci USA* **93**: 8395–8400

Telford WG, King LE and Fraker PJ (1994) Rapid quantitation of apoptosis in pure and heterogenous cell populations using flow cytometry. *J Immunol Methods* **172**: 1

Thornberry NA, Rano TA, Peterson EP, Rasper DM, Timkey T, Garcia-Calvo M, Houtzager VM, Nordstrom PA, Roy S, Vaillancourt JP, Chapman KT and Nicholson DW (1997) A combinatorial approach defines specificities of members of the caspase family and granzyme B. Functional relationships established for key mediators of apoptosis. *J Biol Chem* **272**: 17907–17911

Tsujimoto Y, Cossman J, Jaffe E and Croce CM (1985) Involvement of the *bcl-2* gene in human follicular lymphoma. *Science* **228**: 1440–1443

Wang X, Zelenski NG, Yang J, Sakai J, Brown MS and Goldstein JL (1996) Cleavage of sterol regulatory element binding proteins (SREBPs) by CPP32 during apoptosis. *EMBO J* **15**: 1012–1020

Yang E, Zha J, Jockel J, Boise L, Thompson CB and Korsemeyer SJ (1995) Bad, a heterodimeric partner for Bcl-$X_L$ and Bcl-2, displaces Bax and promotes cell death. *Cell* **80**: 285–291

Yang J, Liu X, Bhalla K, Kim, CN, Ibrado AM, Cai J, Peng T, Jones DP and Wang X (1997) Prevention of apoptosis by Bcl-2: release of cytochrome c from mitochondria blocked. *Science* **275**: 1129–1132

Zaidi SI, Oleinick NL, Zaim MT and Mukhtar H (1993) Apoptosis during photodynamic therapy-induced ablation of RIF-1 tumors in C3H mice: electron microscopic, histopathologic and biochemical evidence. *Photochem Photobiol* **58**: 771–776

 © Cancer Research Campaign 1999
